# Development of Nifedipine Phytantriol-Based Cubosomes and In Vitro Simulation of Administration Through Pediatric Feeding Tubes

**DOI:** 10.3390/pharmaceutics17070828

**Published:** 2025-06-25

**Authors:** Lorena Almeida Lima, Euler Eduardo Lisboa de Moura, Schauana Freitas Fraga, Renata Vidor Contri, Irene Clemes Külkamp-Guerreiro

**Affiliations:** 1 Programa de Pós-Graduação em Ciências Farmacêuticas, Universidade Federal do Rio Grande do Sul, Porto Alegre 90610-000, Brazil; lima.lorealmeida@gmail.com (L.A.L.); schauanafreitas@gmail.com (S.F.F.); renata.contri@ufrgs.br (R.V.C.); 2 Graduação em Química Bacharelado, Universidade Federal do Rio Grande do Sul, Porto Alegre 90610-000, Brazil; euler.moura@ufrgs.br

**Keywords:** pediatric feeding tube, nifedipine, phytantriol nanocarrier, ultrasonication, liquid formulation

## Abstract

**Background/Objectives:** This study focused on developing an organic solvent-free formulation of phytantriol-based cubosomes for nifedipine delivery. It assessed the physicochemical properties and in vitro administration performance in pediatric nasogastric tubes and preliminarily evaluated toxicity in a brine shrimp lethality model. **Methods:** The nanocarrier formulation was characterized in terms of the particle size and drug release properties and was compared with extemporaneous formulations prepared using nifedipine tablets in flow rate tests through pediatric feeding tubes. The recovery efficiency was evaluated across different tube sizes and rinsing volumes. A preliminary toxicity study was conducted using a brine shrimp lethality model. **Results:** Compared with nifedipine tablets, the nanocarrier formulation demonstrated favorable physicochemical properties, including controlled release and superior flow rates, in the pediatric tubes. Full recovery of the nifedipine content was achieved with the nanocarrier formulation, whereas extemporaneous formulation of the nifedipine recovery depended on the tube dimensions and rinsing protocols. **Conclusions:** Compared with the traditional formulations, the nanocarrier formulation represents a promising alternative for administering nifedipine via pediatric feeding tubes, offering an enhanced administration recovery.

## 1. Introduction

Nifedipine is a highly potent calcium channel antagonist belonging to the dihydropyridine group and is widely used in clinical practice to treat angina pectoris and hypertension [1,2,3]. Nifedipine is chemically named the dimethyl ester of 1,4-dihydro-2,6-dimethylpino-4-(2-nitrophenyl)-3,5-pyridinecarboxylic acid. After its administration, nifedipine is almost completely absorbed by the gastrointestinal tract, predominantly from the jejunum; however, it exhibits an extensive first-pass hepatic metabolism, resulting in a reduction in its systemic bioavailability, which is typically approximately 46% to 56%. Nifedipine is classified as a Class II drug in the Biopharmaceutical Classification System and is characterized by high permeability but low solubility, which poses challenges for its formulation. Its low water solubility (~10 μg/mL in water at 37 °C) is associated with poor dissolution, which is identified as one of the causes of its low bioavailability [4,5].

Nifedipine is commonly prescribed to hospitalized pediatric patients, with the dosages varying on the basis of age, weight, and individual needs [6,7,8]. The initial dose for infants older than one month is 0.2–0.5 mg/kg/day. Oral nifedipine has a rapid onset of action (less than 20 min) and can cause a sudden and precipitous drop in blood pressure. Nifedipine is not available in liquid pharmaceutical forms because of its extremely poor water solubility [7,9].

Hospitalized patients often require the use of nasogastric tubes (NGTs) for the administration of nutrients and medications. However, the administration of drugs through pediatric NGTs can be challenging, especially for drugs that lack liquid pharmaceutical formulations. In such cases, solid dosage forms are used and can be manipulated into suspensions by crushing tablets [10]. This tablet-crushing process can result in drug loss, and the prepared suspension may not freely pass through the NGT, leading to potential tube blockage [11].

A promising alternative for developing a liquid pharmaceutical formulation of NIF and enhancing its flow through NGTs is incorporating drugs into nanosystems specifically designed for oral administration. Other hydrophobic drugs, such as spironolactone, have been successfully integrated into nanosystems to create liquid formulations, resulting in neither blockage nor adsorption when these drugs are administered through NGTs [12].

Numerous studies have reported the incorporation of NIF into various types of nanosystems, such as nanoemulsions [13], polymeric nanoparticles [14,15], polymeric micelles [16], and proliposomes [17,18]. In recent years, cubosomes have been explored as nanocarriers because of their potential as efficient drug delivery systems. Cubosomes are nanoparticles of bicontinuous cubic lyotropic liquid crystals composed of curved lipid bilayers containing two internal aqueous channels. Owing to their structure, they can carry lipophilic, hydrophilic, and amphiphilic drugs [19]. Cubosomes also have a high surface area, which makes them capable of incorporating high rates of drugs [20]. The main amphiphilic lipids used in the preparation of cubosomes are phytantriol and glyceryl monooleate. Phytantriol is highlighted as a promising alternative to glyceryl monooleate for the preparation of cubosomes, primarily because of its greater structural stability and reduced susceptibility to degradation by the gastrointestinal tract. Unlike glyceryl monooleate, which is susceptible to esterase-catalyzed hydrolysis, the phytanyl backbone of phytantriol can offer greater structural stability. This stability is crucial for applications such as oral drug delivery, where phytantriol-based cubosomes have demonstrated sustained drug release and improved bioavailability for poorly water-soluble drugs compared with glyceryl monooleate. Additionally, phytantriol dispersions are highly stable during the incorporation of hydrophilic additives and retain the internal Pn3m nanostructure, whereas glyceryl monooleate colloidal dispersions display the HII mesophase, indicating the formation of hexosomes, either alone or in combination with the Pn3m cubic-phase nanostructure of dispersed particles. This makes phytantriol a superior choice over glyceryl monooleate for preparing cubosomes, especially for oral administration and delivery [21,22].

Although many authors have described the advantages of incorporating NIF into nanosystems, no literature works are currently available on applying nanosystems to nifedipine administration through nasogastric tubes. Furthermore, many of the techniques from previous studies have relied on organic solvents, such as chloroform [18] and ethanol [13], to produce nanosystems, raising concerns regarding the presence of toxic residues. Therefore, this study aims to develop an organic solvent-free formulation containing phytantriol-based cubosomes for the incorporation of nifedipine and evaluate it based on physicochemical characterization and in vitro simulation of its administration performance in pediatric nasogastric tubes. A preliminary assessment of toxicity was performed via a brine shrimp lethality model.

## 2. Materials and Methods

### 2.1. The Materials

Nifedipine was obtained from Fagron (Anápolis, Brazil). Phytantriol (3, 7, 11, 15-tetramethyl-1, 2,3-hexadecanetriol, 96.9%) and poloxamer 407 (PEO_98_-PPO_67_-PEO_98_, M_W_ 12,500 g mol^−1^) were purchased from Alianza (São Paulo, Brazil) and Via Farma (São Paulo, Brazil), respectively. Nifedipine tablets (10 mg) were obtained from Neo Química (Anápolis, Brazil), batch B21H2472. The pediatric nasogastric tubes (sizes 4, 6, and 8 FR 100 cm long; polyurethane) were purchased from Mark Med^®^ Santa Apolônia Hospitalar (Florianópolis, Brazil). All reagents were used without any purification process.

### 2.2. Preparation of the NIF-Loaded Nanocarrier

The nanocarriers were prepared as described previously [23], with some modifications. Phytantriol (600 mg) and NIF (2.5 mg) were melted at 40 °C to obtain the oil phase. The aqueous phase (25 mL) containing poloxamer 407 (300 mg) was heated to an equivalent temperature. The solution was homogenized for 25 min using a 350 W ultrasonic processor (QR350W, Ecosonics, São Paulo, Brazil) at a 99% amplitude and using a 13 mm diameter probe. Finally, the final volume was adjusted to 25 mL with deionized water. The cubosomes treated with nifedipine (CN) contained 0.1 mg·mL^−1^ of NIF. This concentration was chosen considering the solubility of nifedipine, as higher concentrations led to the formation of precipitates in the medium. A NIF-free nanocarrier suspension (CB) was also prepared similarly, omitting the drug.

### 2.3. Physicochemical Characterization of the Nanocarriers

#### 2.3.1. Particle Size and Distribution

The diameter and size distribution of the particles were evaluated via the laser diffraction technique (Malvern^®^ 2000 Mastersizer, Malvern Instruments, Malvern, UK). The sample was inserted into a container of distilled water without previous dilution until the desired obscurity was reached. The mean particle size (Z-average) and polydispersity index (PDI) were characterized via dynamic light scattering using a Zetasizer (ZEN 3600 Zetasizer^®^ Nano Series, Malvern Instruments, Malvern, UK) at an angle of 173° at 25 °C. The CB and CN formulations were previously diluted in water (1:100, *w*/*w*). A refractive index of 1.34 was used in both techniques.

#### 2.3.2. The Zeta Potential

The particle surface charge (zeta potential) was determined according to the electrophoretic mobility via a Zetasizer Nano ZS (Malvern Instruments Ltd., Malvern, UK) in triplicate. Measurements were taken immediately after the samples were diluted in deionized water (1:100 *w*/*w*) and previously filtered (0.45 µm, Millipore, Burlington, MA, USA). The zeta potential w expressed in millivolts (mV).

#### 2.3.3. Small-Angle X-Ray Scattering Measurements

Small-angle X-ray scattering (SAXS) measurements were taken to confirm the structure of the nanoparticles via a NanoinXider (XENOCS. Grenobre, France) at CNANO-UFRGS, which operates using a Dectris^®^ Pilatus3 detector and a CuKα X-ray source (λ = 0.154 nm) and is calibrated with a silver behenate standard. The CB and CN formulations were placed into thin-walled boron-rich capillary tubes with an O.D. of 2.0 mm (Charles Supper^®^, Natick, MA, USA). The samples were analyzed without any dilution. Cubosomes are complex liquid crystalline nanoparticles with a unique internal structure. Dilution can alter their morphology, hydration state, and self-assembly characteristics, leading to inaccurate structural interpretations. Consecutive 1 min measurements were obtained over 3 h for each sample in medium-resolution mode at room temperature. The 1D curves were obtained from the 2D SAXS images via Origin^®^ 2019 (OriginLab Corporation, Northampton, MA, USA).

#### 2.3.4. The NIF Content

The NIF content in the nanocarrier suspension was quantified via high-performance liquid chromatography with UV detection (HPLC-UV; PerkinElmer, Waltham, MA, USA). The detection wavelength was 236 nm, the chromatographic column was a C18 (Phenosphere^®^, 150 mm × 4.6 mm × 5 μm, Phenomenex, Torrance, CA, USA), the mobile phase consisted of methanol:water (70:30, *w*/*w*) pH 5 (phosphoric acid), the column temperature was 40 °C, the flow rate was 1.0 mL/min, and the injection volume was 20 μL. The method was evaluated for its specificity, linearity, precision, and accuracy.

#### 2.3.5. The Drug Incorporation Efficiency

The NIF incorporation efficiency (IE) was evaluated via the ultrafiltration-centrifugation method, which is based on principles established in the literature, as described previously [24,25,26]. An aliquot of 400 µL of each formulation was added to filter devices (Microcon 10,000 Da, Millipore, Burlington, MA, USA) and centrifuged at 6000 RPM for 30 min. The drug content in the ultrafiltrate was directly analyzed without prior dilution. The drug IE was expressed as a percentage and was calculated as described in Equation (1):

(1) $$IE(\%)=100\times \frac{C_{T}-C_{I}}{C_{T}}$$

where C_T_ is the total drug content, and C_I_ is the drug content in the ultrafiltrate.

#### 2.3.6. Determination of pH and Density

The pH value was determined by measuring the suspension using a calibrated pH meter (Model DM-22, Digimed, São Paulo, Brazil) via the potentiometric method at room temperature. Density was determined using a glass pycnometer.

#### 2.3.7. The Preliminary Stability Analysis

The CN formulation was subjected to different conditions (room temperature and 40 °C) and protected from light for 28 days. At predetermined times (0, 7, 14, and 28 days), aliquots were obtained from the flasks and evaluated in terms of the mean particle size, PDI, zeta potential, pH, and NIF content, as described in the previous sections.

### 2.4. The In Vitro Release Profile in Simulated Gastric Fluid

The in vitro release was analyzed via an adapted dialysis method (25 mm, MWCO 12 kDa, Sigma–Aldrich, St. Louis, MI, USA). Initially, a dialysis bag containing 2 mL of the formulation was immersed into glass flasks containing simulated gastric fluid (40 mL; pH = 1.2) and maintained at 37 ± 0.5 °C under magnetic stirring (3000 rpm). After 60 min, the dialysis bag was immediately placed into another glass flask with a simulated intestinal fluid pH of 6.8 (a potassium monobasic phosphate mixture and 0.2 M sodium hydroxide; 40 mL), maintained at 37 ± 0.5 °C. At predetermined time intervals, aliquots (1 mL) of the release medium were removed, filtered through a 0.45 μm membrane, and analyzed via HPLC-UV. The removed aliquot volume was replaced with the same volume of fresh release medium. For NIF, the diffusion was tested following the same procedure performed for the CN formulation. The concentration of NIF was quantified via HPLC-UV, as previously described in Section 2.3.4. Three replicates for each experiment were carried out, and the cumulative percentage of the drug release was calculated. The choice of 60 min in the simulated gastric fluid was made on the basis of the theoretical residence time of the formulation in the stomach [27] and on the basis of protocols from previous studies of nanoformulations for the oral route [28].

### 2.5. The Brine Shrimp Mortality Assay

Toxicity was determined via a brine shrimp (*Artemia salina*) lethality bioassay [29] using the CB and CN samples. Initially, *Artemia salina* cysts were placed in vials containing artificial seawater (NaCl 77.23%, MgSO_4_ 9.62%, CaCl_2_ 3.32%, KCl 2.11%, and NaHCO_3_ 0.59%) with a salinity of 38 g/L. After an incubation period of 24 h, the nauplii were separated from the cysts and used for the lethality test [30]. The nauplii were exposed in duplicate to various concentrations of the CB and CN samples, which were prepared through dilution in artificial seawater. The concentrations for testing the CB and CN samples (*w*/*v*) were adjusted on the basis of the concentrations of phytantriol and poloxamer 407 used to prepare the formulations. The following concentrations of the samples were obtained after dilution in artificial seawater: 0.416, 0.208, 0.104, 0.052, 0.026, 0.013, and 0.0065 µg mL^−1^. Ten nauplii were added to each test tube and incubated, with the CB tubes exposed to continuous light and the CN tubes protected to prevent degradation of the active ingredient. Positive control (CTL+) (potassium dichromate) and negative control (CTL−) (artificial seawater) treatments were also performed in parallel to compare the toxicity. Following a 24 h incubation period, the number of surviving nauplii was counted, and the mortality percentages were calculated via the following formula:

Mortality (%) = (number of dead nauplii/total number of nauplii) × 100

where the number of dead nauplii was derived from the difference between the total number of nauplii and the number of surviving nauplii. The LC50 values were then calculated via a probit analysis in GraphPad Prism version 5.0, with the mortality percentages derived from the counts of the surviving nauplii. The toxicity of the formulations was assessed on the basis of the toxicity scales of McLaughlin and Rogers [31]. According to the scale, a lethal concentration for 50% (CL50) value of 1000 µg/mL is considered nontoxic; values between 500 and 1000 µg/mL are considered low-toxicity; values between 100 and 500 µg/mL are considered moderate-toxicity; and finally, when CL50 is below 100 µg/mL, it is considered very toxic.

### 2.6. Preparation of the Extemporaneous Nifedipine Suspensions

Extemporaneous nifedipine suspensions (FE-NIFs) were prepared to simulate the procedure commonly performed in hospitals using the solid form of NIF [10,16]. The suspensions were prepared by crushing 10 mg of nifedipine tablets in a mortar and pestle for 60 s. The theoretical concentration of nifedipine for the suspension was 0.1 mg/mL, the same as that observed for the concentration of nifedipine in the cubosome suspension. The suspensions were used immediately after preparation to assess the passage of the formulations through an NGT.

### 2.7. Evaluation of the Passage of the Formulations Through Pediatric Enteral Nutrition Tubes

To simulate the administration of the formulations (CNs and FE-NIF) through a pediatric NGT, previously reported methodologies were adapted [11,32]

Various 100 cm long pediatric enteral feeding tubes, with calibers of 4, 6, and 8 French (FR), were tested. The tubes were positioned at a 45° angle and kept stationary during administration [33]. All of the tubes were washed with 20 mL of water before the formulations were administered.

Syringes containing the formulations were attached to the ends of the tubes, and the administration was performed slowly to prevent any loss of content during the process. The administration time for all formulations was controlled via a stopwatch to ensure a rate of less than 1.5 mL/s. Importantly, the administration time was established by the authors strictly for experimental control and was not derived from specific pediatric clinical guidelines, institutional protocols, or the specialized literature.

For each formulation (the CNs and the FE-NIF), the experiment was conducted in triplicate, and the samples were collected in a beaker placed at the end of the tube. The syringe containing the FE-NIF was constantly stirred through small rotations during administration to avoid the accumulation of granules on the inner walls of the syringe barrel.

Owing to the limited gastric capacity of infants and newborns [34], the administration volumes for the CN and FE-NIF formulations were defined as 20 mL for the 6 and 8 FR calibers. For the 4 FR probes, the volume was 5 mL since probes of this caliber are intended for newborns.

Between samples, the 6 and 8 FR caliber tubes were rinsed with 2, 5, or 10 mL of purified water to remove residue that occasionally adhered to their walls and to determine the best rinse volume after drug administration. For the 4-FR caliber probe, 1 and 2 mL was used in the rinsing step considering its smaller administration volume.

After passing through the probes, the suspensions (the CN suspension and the FE-NIF) and the rinsing water were collected and analyzed via UV-vis. In brief, 800 µL of the suspensions and the rinsing water were diluted in methanol to obtain a theoretical concentration of 8 µg/mL and subsequently analyzed via UV-vis to quantify the content. The amount of NIF that successfully reached the end of the tube (the amount that would be ingested by the patient) was calculated for all formulations and the rinsing water.

All of the experiments were conducted in triplicate at a controlled temperature of 25 ± 2 °C, which was maintained using an air conditioner and monitored with a room thermometer.

### 2.8. The Statistical Analysis

A paired comparison of the physicochemical characteristics of the CNs and the CB was conducted via paired *t* tests. This parametric test was chosen to assess significant differences between two related sets of measurements. A one-way analysis of variance (ANOVA), followed by Tukey’s post hoc test, was employed to compare the total drug recovery from the FE-NIF and CNs using different probe calibers (4, 6, and 8 FR). An ANOVA was selected because it is appropriate for comparing the means of three or more independent groups, which corresponded to our three distinct probe caliber sizes. Tukey’s post hoc test was subsequently used for multiple comparisons to pinpoint specific significant differences between the individual group means while controlling for the familywise error rate. The significance level for all tests was set at *p* < 0.05, and the analysis was performed via GraphPad Prism version 5.0.

## 3. Results

### 3.1. Physicochemical Characterization

The cubosome formulation was prepared and characterized (Table 1) via the top-down technique using ultrasound equipment. The nanocarriers had particle sizes of 152 ± 5.5 nm (as measured using laser diffraction) and 159 ± 2.8 nm (as measured using dynamic light scattering). The sample showed a homogenous pattern with a Span value of 1.22 ± 0.02 and a PDI of 0.099 ± 0.001. The zeta potential of −18.9 ± 0.78 mV indicated colloidal stability, primarily due to steric effects [35]. The negative charge was attributed to the ionization of the carboxylic acid group in phytantriol [36].

The assay method exhibited excellent specificity for the quantification of nifedipine, enabling its detection independently of the other components of the formulation, such as phytantriol and the surfactant poloxamer 407. The method demonstrated a linear range of 4–12 μg/mL, with a high R^2^ value of 0.9995. The precision analysis revealed repeatable results with relative standard deviations of less than 5% for both the intraday and the interday analyses. The accuracy test revealed an average recovery rate of 99.38 ± 1.72%. The CN sample had a content of 99 ± 0.69% and a high IE of 94.73 ± 4.9%. The pH of the CN sample was close to neutral (6.64 ± 0.12), and its density was similar to that of water (1.050 ± 0.0014 g/mL). The average values for the particle size, PDI, zeta potential, pH, and density of the CN sample were almost identical to those of the CB sample (Table 1). These data demonstrate that the incorporation of the drug molecules into the nanosystem had no significant effect on the analyzed physicochemical parameters compared with those of the drug-free nanosystem (CB).

**Figure 1:**
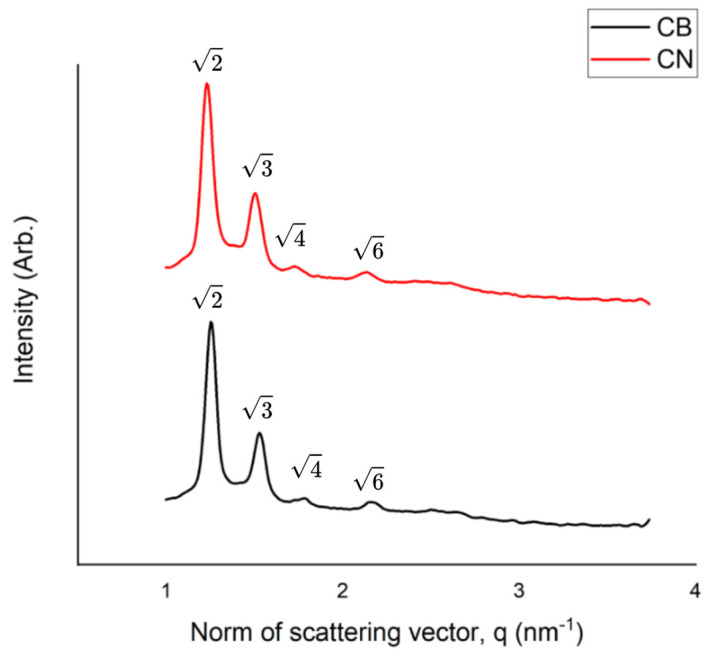
The intensity vs. the norm of the scattering vector obtained via SAXS measurements for the NIF-loaded cubosomes (CNs) and the blank cubosome suspension (CB). q = 2 sinθ/λ, where θ is the Bragg angle and λ is the X-ray wavelength.

The SAXS measurements are shown in Figure 1. The diffraction patterns for both the CB and the CNs showed at least four Bragg peaks with their relative positions at spacing ratios of √2: √3: √4: √6.

**Figure 2:**
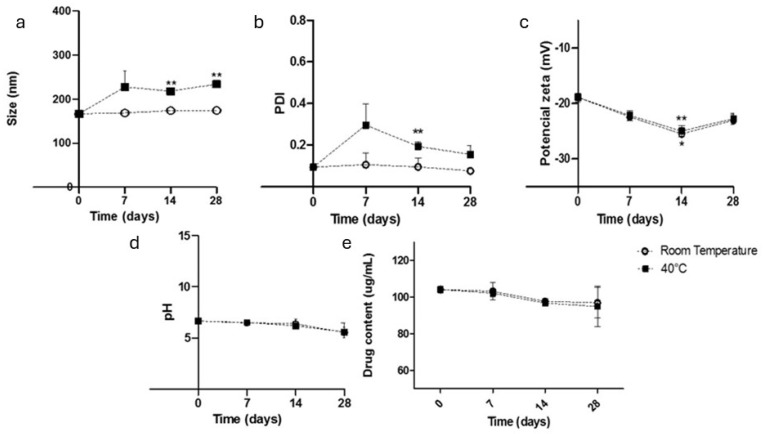
Characterization of the nanocarriers containing nifedipine (CN): mean particle size (**a**), polydispersity index (PDI) (**b**), zeta potential (**c**), pH (**d**), and nifedipine content (**e**) (* refers to *p* < 0.05, ** refers to *p* < 0.01).

No significant changes in the sample homogeneity were observed during storage at either room temperature or 40 °C for 28 days (Figure 2). The samples remained milky white emulsions without aggregation. The samples stored at room temperature presented stable particle sizes, PDIs, pH values, and contents over the 28-day period. The zeta potential increased significantly after 14 days but did not indicate particle degradation. However, the samples stored at 40 °C presented variations in particle size and PDI [21].

After 28 days of storage, the NIF content in the samples decreased by 10% compared with the initial concentration. This degradation is attributed to the hydrolysis reactions of the ester groups present in NIF when it is exposed to an aqueous environment [37,38]. The present results for the NIF cubosomes proved to be more advantageous than previous reports on nifedipine polymeric nanoparticles subjected to different storage temperature conditions (25 °C and 40 °C), where at the end of 30 days, there was a significant increase in the particle size (134.8 nm to 273.8 nm at 25 °C and 681.8 nm at 40 °C) and significant variations in the PDI (0.08 to 0.419 at 40 °C and 0.255 at 25 °C) [13].

### 3.2. In Vitro Release

**Figure 3:**
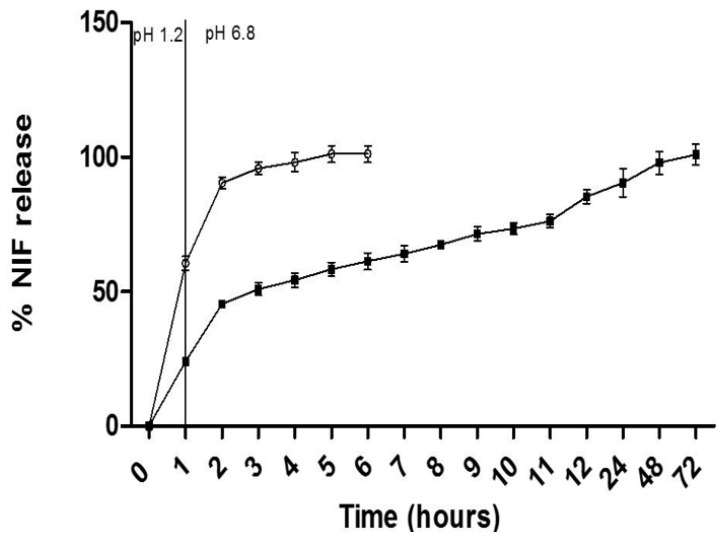
The NIF release profile for the CN 0.1 mg/mL (black squares) and free NIF 0.1 mg/mL (white circles) in simulated gastric and intestinal fluid media. The data are expressed as the percentage of NIF versus time (hours).

The NIF release in both the free drug dispersion and the CN formulation varied over time and was influenced by the simulated fluid medium (Figure 3). In the simulated gastric medium (pH = 1.2), the dispersion of free NIF released 60.53 ± 2.58% in 60 min, whereas the CN formulation promoted a delay in the release of NIF, releasing 24.05 ± 1.1% in 60 min. These results demonstrate that the CN formulation has the ability to trap NIF in the system at a simulated gastric pH.

Compared with free nifedipine, the nanocarriers resulted in a lower rate of release of nifedipine in the simulated intestinal fluid, indicating a controlled release effect (24.05 ± 1.1% and 60.53 ± 2.5%, respectively). After 6 h, the formulation with free NIF released 100% of the drug, while the CN formulation released 61.23 ± 3.05%. Complete release of NIF from the nanocarriers was observed only after 72 h.

### 3.3. The Passage of the Formulations Through Pediatric Enteral Nutrition Tubes

The flow rate of the formulations through the tubes varied depending on the formulation type and the tube caliber. No obstructions were observed during the study; however, greater resistance was observed in the passage of the extemporaneous formulation (FE-NIF) for the 4-FR caliber probes. The improved flow rate of the CN sample compared with that of the FE-NIF can be attributed to the lower viscosity of the colloidal system compared with that of the extemporaneous formulation. Therefore, it can be assumed that less force is required for the administration of the nanoparticle suspensions through the probe, which facilitates a continuous flow [39].

The recovery percentages of NIF from the CNs and FE-NIF after rinsing with different volumes of the 4, 6, and 8 FR caliber tubes are presented in Table 2. The rinsing water volume used in the 4 FR caliber probes did not have a statistically significant effect on the total recovery of NIF for the extemporaneous formulations (FE-NIFs). The drug recovery values were significantly lower, almost 30% less than the content of the administered sample, indicating the poor reliability of the tablet-crushing method of administering the desired dosage to the patient. The total NIF recovery content increased according to the caliber used to administer the FE-NIF. After passing through the FE-NIF, the recovered NIF content was greater for the 8 FR probe than that for the 6 FR probe, indicating that a larger caliber facilitated the passage of the dispersion. The statistical analysis revealed that rinsing the probe with a volume of 10 mL significantly improved the total NIF recovery content. Although the rinsing volume increased the FE-NIF drug recovery for the 6 and 8 FR caliber probes, they were still not equivalent to the original dosage of the NIF tablets. The CN formulation, however, presented complete drug recovery independent of the rinsing volume used, indicating its delivery efficiency for all probe calibers.

### 3.4. The Brine Shrimp Mortality Assay

**Figure 4:**
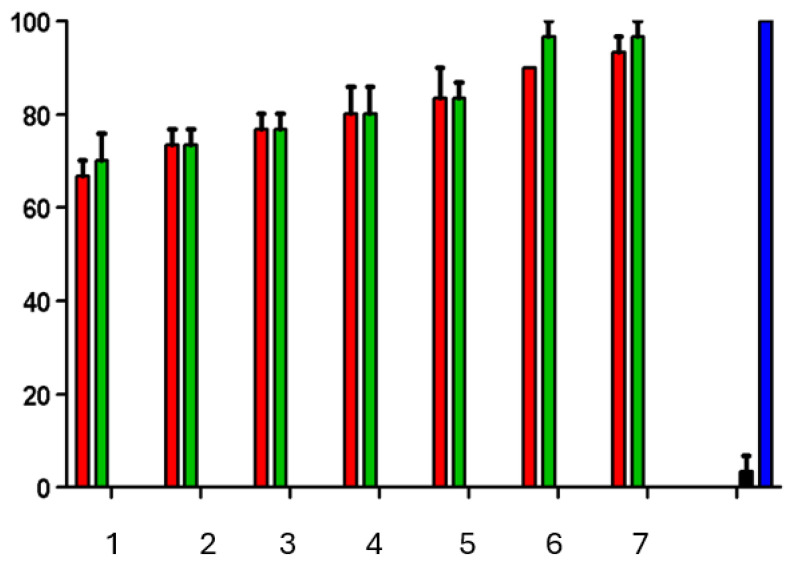
*Artemia salina* viability (%) in y axis versus concentration of the CN (red) and CB (green) formulations, positive control group potassium dichromate (black), and negative control group saline seawater (blue). Concentrations are 0.416 (1), 0.208 (2), 0.104 (3), 0.052 (4), 0.026 (5), 0.013 (6), and 0.0065 (7) µg/mL.

The results in terms of *Artemia salina* lethality for the CB and CN formulations indicated that lethality was influenced by the concentration of the tested formulations, with higher concentrations resulting in increased lethality (Figure 4). The positive control using potassium dichromate showed lethality close to 100% after 24 h, whereas the negative control using saltwater had no lethal effect. The LC_50_ (the lethal concentration for 50% of the samples) was determined for the CB and CN formulations, which demonstrated the low toxicity of both formulations. Previous studies using different types of nanoparticles have also shown toxic effects at different stages of *Artemia salina* development depending on the type of nanoparticle used.

## 4. Discussion

The results of the physicochemical characterization demonstrated that the nifedipine-containing cubosome (CN) formulation presented the appropriate physicochemical properties, with a nanometric particle size, low PDI values, and a negative zeta potential, suggesting its good colloidal stability. Furthermore, it showed a high drug IE without significant changes in the physical parameters compared with those of the control formulation (CB), implying that the presence of nifedipine did not compromise the system’s structure.

Similarly high IE values (93%) were reported with the incorporation of NIF into monoolein cubosomes [18]. In contrast, lower IE values were observed for NIF incorporated into polymeric micelles (34.1 ± 1.1%) [16] and poly(D,L-lactide-co-glycolide) (61.81 ± 3.41%) [40]. The high efficiency of incorporating NIF into the cubosomes can be explained by the high lipophilicity of the drug, promoting complete solubilization of NIF by the oil phase.

The incorporation of the drug NIF into monoolein-based cubosomes was investigated previously, aiming to improve its oral bioavailability [18]. The particle size obtained was 91.3 nm, the PDI was 0.168, and the zeta potential was −12.8 mV. However, this cubosome production process involves the use of the organic solvent chloroform, raising concerns regarding the presence of toxic residues. Additionally, the processing time required for sample production was long, exceeding 24 h.

In contrast, in the present study, cubosomes were developed via a solvent-free production approach, with a significantly reduced processing time of only 25 min. This approach mitigates the risks associated with the presence of organic solvent residues in the samples and enhances the safety of the final product.

The three possible structures of the cubic phase are Pn3m, Im3m, and Ia3d [21]. The peaks in the SAXS pattern for the CB and CNs can be indexed according to the Miller indices hkl = 110, 111, 200, and 211, which are indicative of the cubic phase Pn3m [41,42].

In previous studies, the release profile of NIF when incorporated into proliposomes was lower than that of free NIF, similar to that of the cubosomes. However, the cumulative release of NIF from the proliposomes reached 88.7% in artificial intestinal fluid [17], whereas in the present study, after 10 h, the cumulative release of NIF from the cubosomes was 73.47 ± 2.17%, indicating that incorporating NIF into cubosomes resulted in a slower release rate than that under its incorporation into proliposomes. When NIF is incorporated into monoolein cubosomes, the rate of release of NIF is approximately 5% for both simulated gastric fluid and simulated intestinal fluid over a period of 24 h [18].

Another advantage of the present system is the use of phytantriol, instead of monoolein, as the structural component of the cubosomes. While the properties of both have been detailed in previous reports [21,22], monoolein has been reported to exhibit gastric instability, whereas phytantriol has greater resistance to the gastric environment, making it more suitable for oral administration of the drug. This contrast in stability is a key factor in ensuring the efficacy of NIF upon oral administration [21,22].

In summary, the present study overcomes these limitations by offering a more efficient approach to the production of cubosomes that are free from organic solvents and that use phytantriol as a gastric-resistant structural component. The in vitro results indicate potential for future studies on the clinical application of these nanosystems to oral drug delivery.

The impact of rinse volume on the total recovery of proton pump inhibitors in pediatric tubes has been studied previously [11]. Similarly, the authors reported that higher rinse volumes led to greater recovery percentages. In the present study, the CN sample presented a 100% recovery rate for all probes, with no losses during administration. This result can be attributed to the high IE when nifedipine was incorporated into the cubosomes. Administering medications through feeding tubes poses challenges in the hospital setting, emphasizing the importance of considering the formulation characteristics. Significant losses were observed during the administration of the extemporaneous formulations, and tube rinsing protocols are commonly used to minimize losses. Grinding methods, such as using a mortar and pestle, have resulted in losses during the crushing process for paracetamol.

Another report in the literature reported that the process of crushing a sotalol tablet in a mortar and transferring it into another container resulted in a loss of 5.5–13% of the tablet’s weight [43]. This finding, along with our results, suggests that the losses during the preparation process for an extemporaneous formulation can vary and significantly affect the total percentage of NIF recovered at the end of its administration through an FE-NIF.

One consequence of losses in the total percentage of FE-NIFs is the administration of inconsistent doses. Additionally, drug residues in nasogastric tubes can interact with food, leading to the formation of bezoars, which can cause tube blockage [44]. To date, no studies have reported the oral bioavailability of crushed NIF tablets administered via nasogastric tubes. This lack of information combined with the high percentage of NIF lost during the administration process further increase the uncertainty surrounding this practice.

The greater resistance observed in the passage of the extemporaneous formulation (FE-NIF) has significant clinical implications. This increased resistance could translate into longer administration times; increased nursing workloads and patient discomfort; the need for increased force during administration, potentially leading to tube damage or patient injury; and a greater risk of tube blockage [11,32]. Moreover, the incomplete drug recovery observed with the extemporaneous formulation, even with rinsing, underscores the inaccuracy of this administration method and the potential for underdosing, further supporting the need for alternative formulations [11].

In contrast, the CN nanotechnology formulation shows promise in overcoming these limitations. The total recovery rate for the CN sample was 100%, regardless of the probe size or the rinse volume. The total percentage of NIF recovery for the FE-NIF was influenced by the probe caliber and the rinse volume.

Unlike what has been observed for other nanoparticles, the CN sample showed a safe profile in the *Artemia salina* assay. Studies using gold nanoparticles (AuNPs), silver nanoparticles (AgNPs), and titanium dioxide nanoparticles (TiO₂NPs) have demonstrated the broad sensitivity of *Artemia salina* at different developmental stages, depending on the type of nanoparticle used [45]. Exposure to different nanoparticles at concentrations of 10⁻^1^, 10⁻^2^, and 10⁻^3^ μg/mL revealed that the TiO₂NPs were nontoxic, that the AuNPs had toxic effects on cyst hatching but not on nauplii development, and that the AgNPs were toxic to both hatching nauplii and adults.

This in vitro study has inherent limitations, as it cannot fully replicate the complex physiological environment and variability observed in patient populations. Factors such as individual differences in gastrointestinal pH, enzyme activity, and transit time are not accounted for in our model. Moreover, our in vitro simulation fails to encompass the range of real-world administration conditions, including variations in the feeding tube types, administration rates, and potential interactions with enteral nutrition. Significant limitations of this study include the absence of in vivo data on bioavailability and systemic safety, the exclusive reliance on in vitro models and enteral administration simulations, and a reduced sample size, which restricts the generalizability of the results.

## 5. Conclusions

A new phytantriol-based cubosome formulation containing nifedipine was developed without the use of organic solvents, suggesting its potential suitability for pediatric use, pending further in vivo validation. The formulation presented physicochemical parameters compatible with those of the cubosomes. The in vitro data from the CN formulation suggest the possibility of its administration through enteral nutrition tubes, with a total recovery rate of 100% for all tested probe sizes (4, 6, and 8 FR). Furthermore, the rinsing volume did not affect the recovery rate of NIF for the CN sample, indicating that a minimal rinsing volume could be used during probe flushing. These results indicate that the CN formulation has the potential to overcome common issues encountered in hospitals during the administration of crushed nifedipine tablets via enteral feeding tubes, such as tube blockage. The CN formulation has the potential to prevent a reduction in the active ingredient during the preparation and administration of formulations through enteral nutrition tubes.

The findings from this study are limited to in vitro conditions and necessitate further validation through in vivo testing. Future research should encompass thorough in vivo pharmacokinetic and pharmacodynamic assessments, extended safety evaluations, and detailed toxicological analyses to confirm the appropriateness of the formulation for pediatric use. To achieve this validation, in vivo studies should evaluate the performance of the formulations in relevant animal models or ultimately in clinical trials. These studies should incorporate pharmacokinetic and pharmacodynamic assessments. Furthermore, future investigations should evaluate the interaction of the formulations with various enteral feeding solutions and conduct further investigations into their performance under simulated physiological conditions that mimic the variations observed in patient populations better.

## Figures and Tables

**Table 1:** Physicochemical characterization of the CB and CN (0.1 mg/mL) formulations.

	Sample	
	CB	CN
D [4, 3] (v) (nm)	145 ± 6.1 ^a^	152 ± 5.5 ^a^
Span (v)	1.22 ± 0.02 ^a^	1.22 ± 0.02 ^a^
Size (nm)	156 ± 0.17 ^a^	159 ± 2.8 ^a^
PDI	0.099 ± 0.001 ^a^	0.094 ± 0.018 ^a^
Zeta potential (mV)	−17.3 ± 0.55 ^a^	−18.9 ± 0.78 ^a^
pH	6.79 ± 0.07 ^a^	6.64 ± 0.12 ^a^
Density (g/mL)	1.050 ± 0.0014 ^a^	1.020 ± 0.001 ^a^
Drug content (%)	-	99 ± 0.69
IE (%)	-	94.73 ± 4.9

The results are expressed as the means ± standard deviations. Matching letters within the same row indicate no significant difference (*p* ≤ 0.05).

**Table 2:** NIF recovery after passage through 4, 6, and 8 FR caliber pediatric tubes with different rinse volumes.

			Total Drug Recovered (%) After Passing Through the Probe			
Probe Caliber	Sample	Original Drug Content (%)	Probe 1 (1 mL Rinse)	Probe 2 (2 mL Rinse)	Probe 3 (5 mL Rinse)	Probe 4 (10 mL Rinse)
4 FR	FE-NIF	101.1 ± 1.8 ^a^	62.5 ± 4.2 ^b^	67.6 ± 5.6 ^b^	—	—
	CN	102.5 ± 4 ^a^	103.9 ± 4 ^a^	100.3 ± 4 ^a^	—	—
6 FR	FE-NIF	101.1 ± 1.8 ^a^	—	65.0 ± 12 ^b^	72.6 ± 6 ^b^	82.9 ± 9 ^b^
	CN	102.5 ± 4 ^a^	—	103.9 ± 4 ^a^	100.3 ± 4 ^a^	100.8 ± 5 ^a^
8 FR	FE-NIF	101.1 ± 1.8 ^a^	—	70.2 ± 9.3 ^b^	78.1 ± 8.9 ^b^	91.1 ± 10 ^a,b^
	CN	102.5 ± 4 ^a^	—	103.9 ± 4 ^a^	100.3 ± 4 ^a^	100.8 ± 5 ^a^

Results expressed as the mean ± standard deviation; matching letters within a column indicate no significant difference (*p* ≤ 0.05).

## Data Availability

The raw data supporting the conclusions of this article will be made available by the authors on request.

## Abbreviations

The following abbreviations are used in this manuscript:

CNs	Cubosomes with Nifedipine
CB	Control Blank
FE-NIF	Extemporaneous Formulation of Nifedipine
NGT	Nasogastric Tube
IE (%)	Incorporation Efficiency
PDI	Polydispersity Index
SAXS	Small-Angle X-Ray Scattering
CL50	Lethal Concentration for 50%
CTL+	Positive Control
CTL−	Negative Control
FR	French
